# Assessing Family Functioning Before and After an Integrated Multidisciplinary Family Treatment for Adolescents With Restrictive Eating Disorders

**DOI:** 10.1192/j.eurpsy.2022.1485

**Published:** 2022-09-01

**Authors:** C. Rogantini, M. Orlandi, L. Provenzi, M. Chiappedi, C. Coci, M. Criscuolo, M. Castiglioni, V. Zanna, R. Borgatti, M. Mensi

**Affiliations:** 1 University of Pavia, Department Of Brain And Behavioural Sciences, Pavia, Italy; 2 Fondazione Mondino - Istituto Neurologico Nazionale IRCCS, Child Neuropsychiatry, Pavia, Italy; 3 Bambino Gesù children hospital IRCCS, Anorexia Nervosa And Eating Disorders Unit, Roma, Italy

**Keywords:** Adolescents, Integrated Multidisciplinary Family Treatment, Restrictive Eating Disorders, Family functioning

## Abstract

**Introduction:**

Previous studies applying the Lausanne Trilogue Play (LTPc), a semi-structured method for observing family dynamics, highlighted dysfunctional interaction patterns in the families of individuals affected by restrictive eating disorders (REDs). Family-centered approaches are considered the first-line treatment for severe cases of REDs in adolescence.

**Objectives:**

To investigate family functioning in the families of adolescents with severe REDs assessed before and 6 months after a multidisciplinary family treatment program that combined psychodynamic psychotherapy, parental role intervention and triadic or family-centered intervention.

**Methods:**

Sixty-seven families of adolescent patients diagnosed with REDs were assessed for eligibility between July 2017 and October 2020. Family functioning was assessed using the clinical version of LTPc. Nutritional counseling and neuropsychiatric monitoring were also provided.

**Results:**

We observed a significant change in the family functioning score for the LTPc phase 2, in which the father interacts with his daughter while the mother acts as a silent observer. This suggests that the fathers, when playing an active role, could improve dyadic family functioning. The treatment was not found to change triadic functioning: a 6-month treatment may not be long enough to modify interactions at the triadic level.

**Conclusions:**

A brief multidisciplinary treatment program may significantly improve family functioning in the families of patients diagnosed with severe REDs. Although appropriate clinical trials are needed to further test the efficacy of this treatment, our study reinforce the concept that treatment programs targeting the individual patient and both the parents should be a first-line approach in adolescents with severe REDs.

**Disclosure:**

The authors declare that they do not have a significant financial interest, consultancy or other relationship with products, manufacturer(s) of products or providers of services related to this abstrac.

